# Supplementary material for: Guanine crystal formation by bacteria
Source: BMC Biol. 2023 Apr 3;21:66. doi: 10.1186/s12915-023-01572-8 (PMC10071637; doi:10.1186/s12915-023-01572-8)
Supplement: Supplementary file 1 — Additional file 1: Figure S1. Crystalline aggregates in colonies from a month-old plate of A. salmonicida subsp. pectinolytica 34mel. Figure S2. Size distribution of the base of the prismatic crystals formed by 34mel grown in different conditions and by A. media. [file 12915_2023_1572_MOESM1_ESM.pdf]

Supplementary Information for

Guanine crystal formation by bacteria

María Elisa Pavan, Federico Movilla, Esteban E. Pavan, Florencia Di Salvo, Nancy I. López, M. Julia Pettinari

Additional file 1

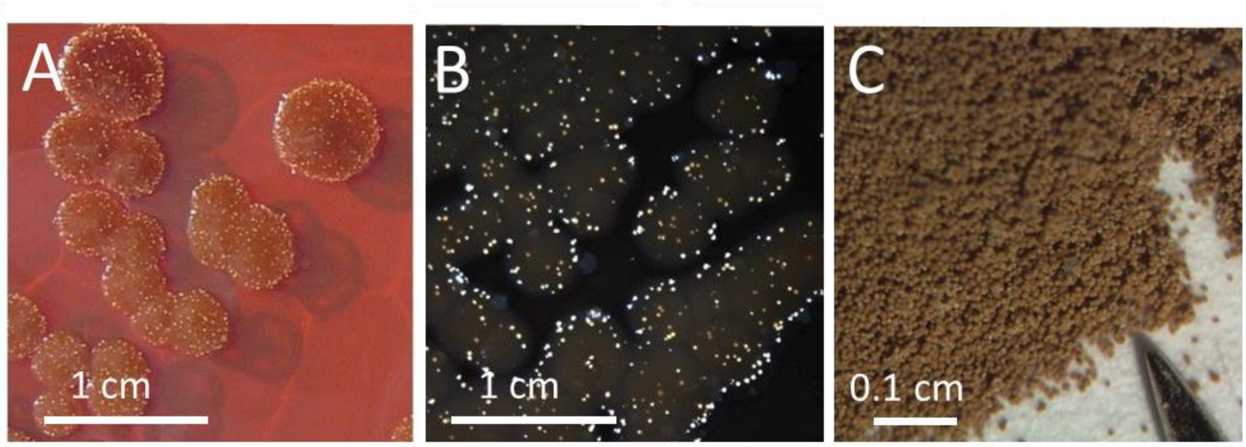

Fig. S1. Crystalline aggregates in colonies from a month-old plate of *A. salmonicida* subsp. *pectinolytica* 34mel. A Bacterial colonies displaying the brown color due to melanin production. The crystal aggregates are seen as bright particles. **B** Bacterial colonies seen under polarized light microscopy revealing the birefringence of the crystalline aggregates. **C** Guanine crystals collected from several plates. The tip of a common pin is shown for size comparison.

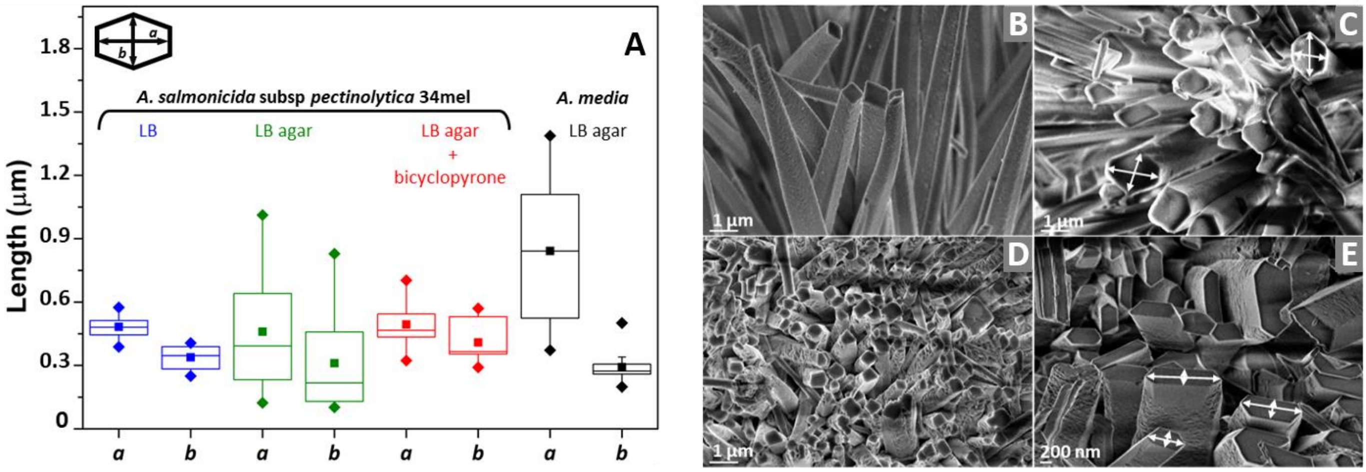

Fig. S2. Size distribution of the base of the prismatic crystals formed by 34mel grown in different conditions and by *A. media*. A The base of individual crystals was measured for each experimental condition (n=12) and data analyzed using ImageJ [39].The box limits represent the range between the first and third quartiles for each condition, the center lines show the median, the square symbols represent the mean, and the ends of the whiskers extend to 1.5× the interquartile range. The diagram in the top left corner shows the long (a) and short (b) axis used for size analysis. **B-E** SEM micrographs of the biogenic crystals of 34mel grown on liquid culture (**B**), agar plates (**C**), agar plates with the inhibitor bicyclopyrone (**D**) and *A. media* in agar plates (**E**).
