# Supplementary material for: Guanine crystal formation by bacteria
Source: BMC Biol. 2023 Apr 3;21:66. doi: 10.1186/s12915-023-01572-8 (PMC10071637; doi:10.1186/s12915-023-01572-8)
Supplement: Supplementary file 2 — Additional file 2: Figure S3. ESI–MS experiment for guanine produced by 34mel. Figure S4. ESI–MS and MS/MS experiments for commercial guanine. Figure S5. 1H-NMR characterization of 34mel crystals. Figure S6. UV–vis spectra for commercial and 34mel guanine in acid solution. Additional data 1. Elemental analysis of crystals purified from 34mel. [file 12915_2023_1572_MOESM2_ESM.pdf]

Supplementary Information for

Guanine crystal formation by bacteria

María Elisa Pavan, Federico Movilla, Esteban E. Pavan, Florencia Di Salvo, Nancy I. López, M. Julia Pettinari

Additional file 2

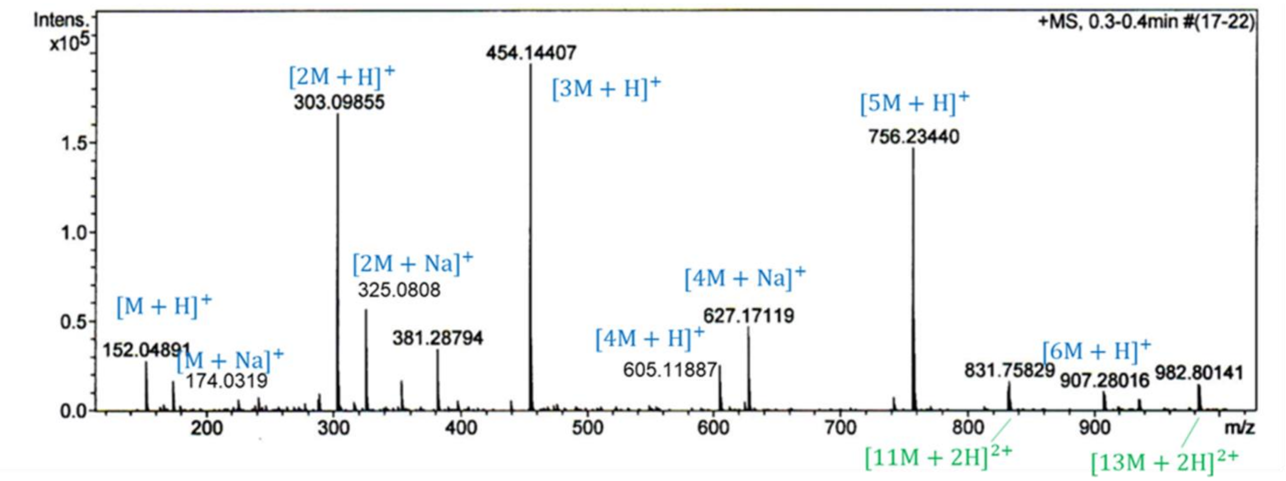

Fig. S3. ESI-MS experimental results for guanine produced by 34mel. The spectrum shows guanine association in solution. Solvent: methanol: DMSO 80:20.

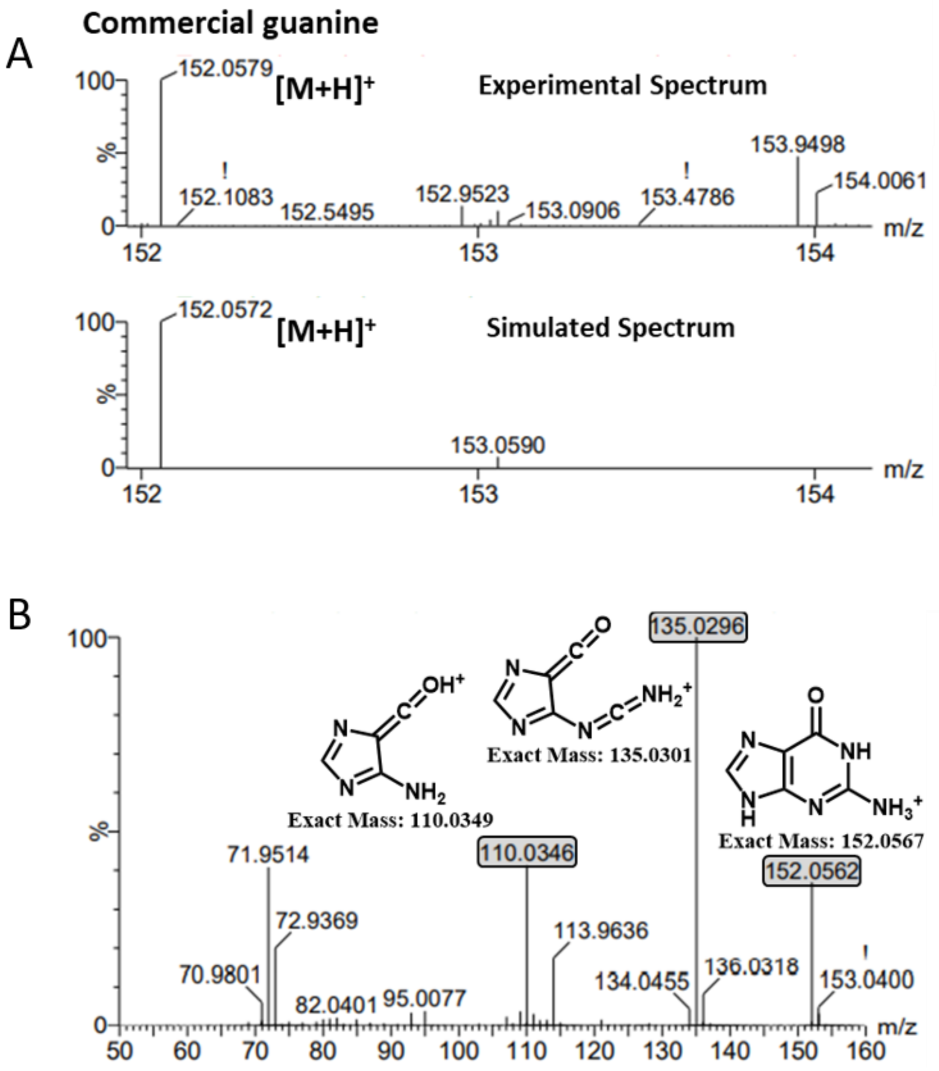

Fig. S4. ESI-MS and MS/MS experiments for commercial guanine. A ESI-MS experimental result compared with the simulated spectrum; $[M+H]^+$ ion (m/z 152.0579) is indicated. **B** MS/MS experimental results using $[M+H]^+$ ion (m/z 152.0579) as the parent ion. Solvent: methanol: H_2O.

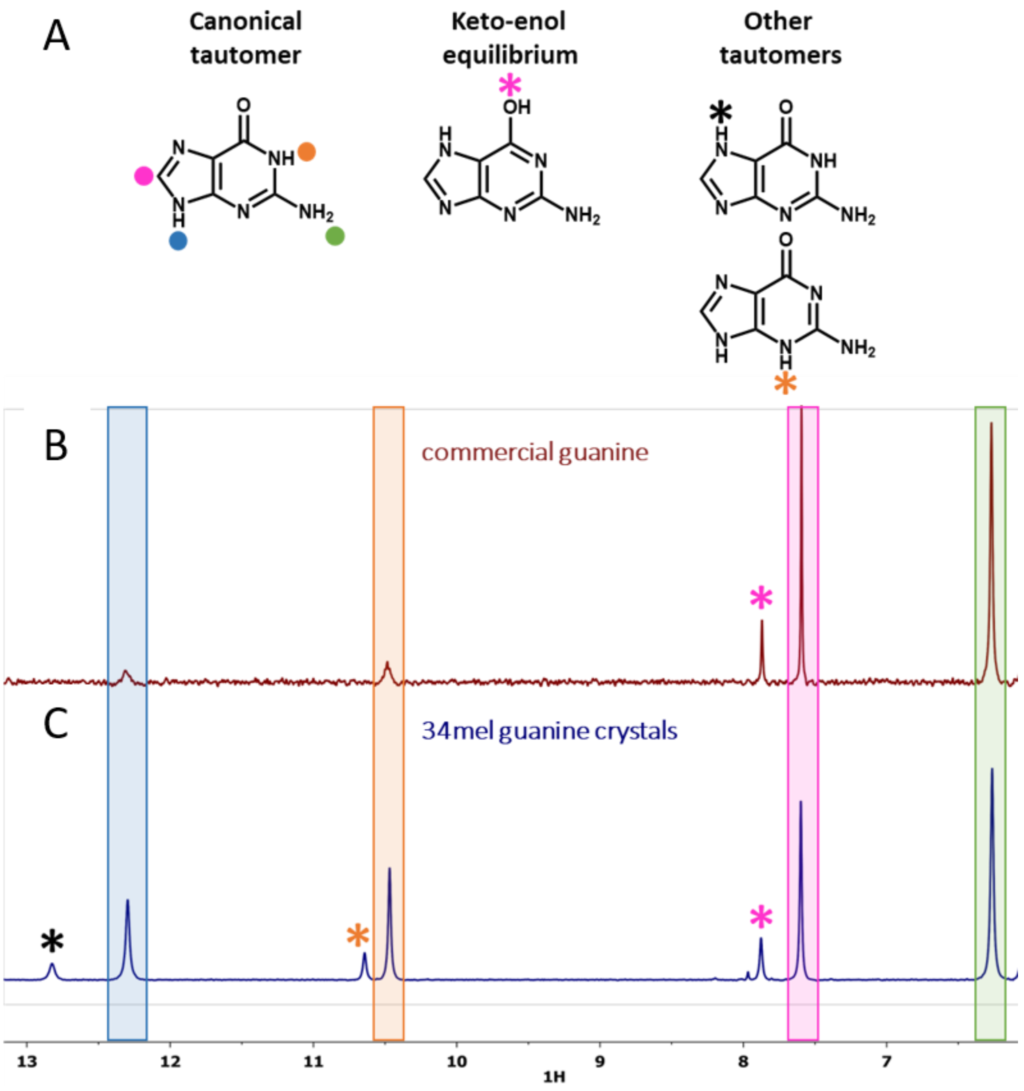

Fig. S5. ^{1}**H-NMR characterization of 34mel crystals. A** Scheme of some of the expected tautomeric forms of guanine. **B-C** ^{1}H-NMR spectra for commercial guanine (**B**) and guanine crystals produced by 34mel (**C**). In color, signal assignment for guanine hydrogens and proposed signal assignment that may be produced from tautomers in solution.

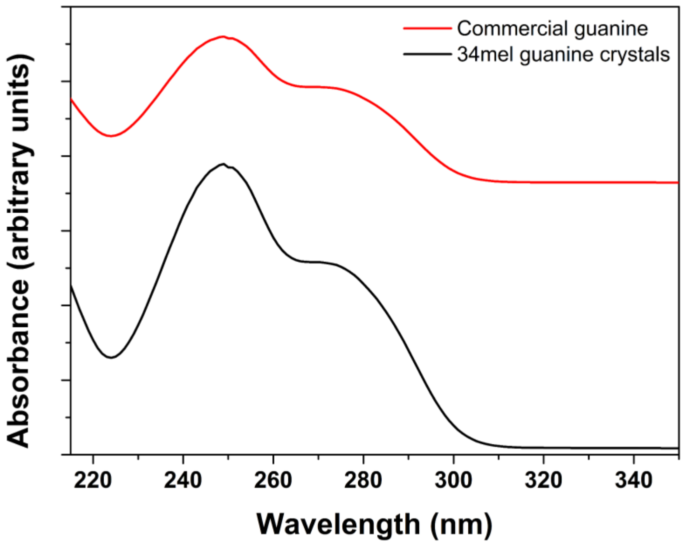

Fig. S6. UV-vis spectra for commercial and 34mel guanine in acid solution.

Additional data 1. Elemental analysis of crystals purified from 34mel.

The presence of biogenic melanin and water molecules were taken into account in the calculated values of C% H% and N% for the guanine samples. Biogenic melanin was represented using a pyomelanin monomer which corresponds to a homogentisic acid unit with two hydrogen atoms removed [$(C_8H_4O_4)_n$]. A good agreement was obtained when considering 6% of the monomer and 14% of water [$(C_5H_5N_5O)_{15}(C_8H_4O_4).22H_2O$].
