# Supplementary material for: Guanine crystal formation by bacteria
Source: BMC Biol. 2023 Apr 3;21:66. doi: 10.1186/s12915-023-01572-8 (PMC10071637; doi:10.1186/s12915-023-01572-8)
Supplement: Supplementary file 3 — Additional file 3: Additional methods 1. Methods used for the synthesis of the guanine crystalline phases from commercial guanine. Figure S7. X-ray diffraction studies of different crystal forms of guanine. Figure S8. Crystalline material obtained as result of crystallization experiments of commercial guanine with or without the addition of homogentisate melanin synthesized by 34mel at different pH conditions. Figure S9. Powder X-ray diffraction experiments of the crystalline material obtained under different crystallization conditions. Additional methods 2. Single crystal X-ray diffraction (XRD) of guaninium chloride dihydrate-melanin. Figure S10. Structure of guaninium chloride dihydrate crystallized in presence of melanin produced by the 34mel determined by single crystal X-ray diffraction. Table S1. Crystal data and structure refinement for guaninium chloride dihydrate crystallized in presence of melanin produced by 34mel. Table S2. Comparative analysis of different guaninium chloride dihydrate single crystal X-ray diffraction data. [file 12915_2023_1572_MOESM3_ESM.pdf]

Supplementary Information for

Guanine crystal formation by bacteria

María Elisa Pavan, Federico Movilla, Esteban E. Pavan, Florencia Di Salvo, Nancy I. López, M. Julia Pettinari

Additional file 3

Additional methods 1

Synthesis of the guanine crystalline phases using commercial guanine

For crystallization of guanine at basic pH, crystals were produced by adding 20 mg of guanine powder (Sigma−Aldrich) in 600 μL of pure water. The solid was completely dissolved after dropwise addition of a solution of NaOH 1M, adjusting the pH of the solution to a value of 10. The resulting clear solution was left open to the air at room temperature until white crystalline solids were observed. Crystalline material was then collected by centrifugation, washed several times with distilled water and finally dried under vacuum. To obtain the crystals with melanin, filtered melanized liquid culture was used to prepare the starting solution instead of water. As a result, a brown crystalline solid was obtained.

For crystallization of guanine at acid pH, crystals were produced by adding 20 mg of guanine powder (Sigma−Aldrich) in 600 μL of pure water and then, the solid was partially dissolved after dropwise addition of a solution of HCl 1M adjusting the pH of the solution to a value of 2. The resulting suspension was sonicated for 5 minutes and then heated at 90°C for an hour, to obtain a clear solution. Crystalline material was then collected by centrifugation, washed several times with distilled water and finally dried under vacuum. To obtain the crystals with melanin, filtered melanized liquid culture was used to prepare the starting solution instead of water. Due to the low pH, biogenic melanin remained partially soluble and guanine was not fully dissolved after sonication and heating. Therefore, the resulting turbid solution was filtered before crystallization. Crystalline material was then collected by centrifugation, washed several times with distilled water and finally dried under vacuum. As a result, a pale-brown crystalline solid was obtained.

Guaninium chloride dihydrate crystals were produced by adding 20 mg of guanine powder (Sigma−Aldrich) in 500 μL of pure water and then 200 μL of HCl (c) was added (pH ~ 0). The resultant suspension was warmed until complete dissolution of the guanine. The resulting clear solution was left open to the air at room temperature until white elongated needle-like crystals were observed. Crystalline material was then collected by centrifugation, washed several times with distilled water and finally dried under vacuum. To obtain the crystals with melanin, filtered melanized liquid culture was used to prepare the starting solution. Due to the low pH, biogenic

melanin remained partially soluble and guanine was not fully dissolved after sonication and heating, so the resultant turbid solution was filtered before crystallization. Crystalline material was then collected by centrifugation, washed several times with distilled water and finally dried under vacuum. As a result, pale-brown elongated needle-like crystals were obtained; this material resulted to be suitable for single crystal X-ray diffraction studies.

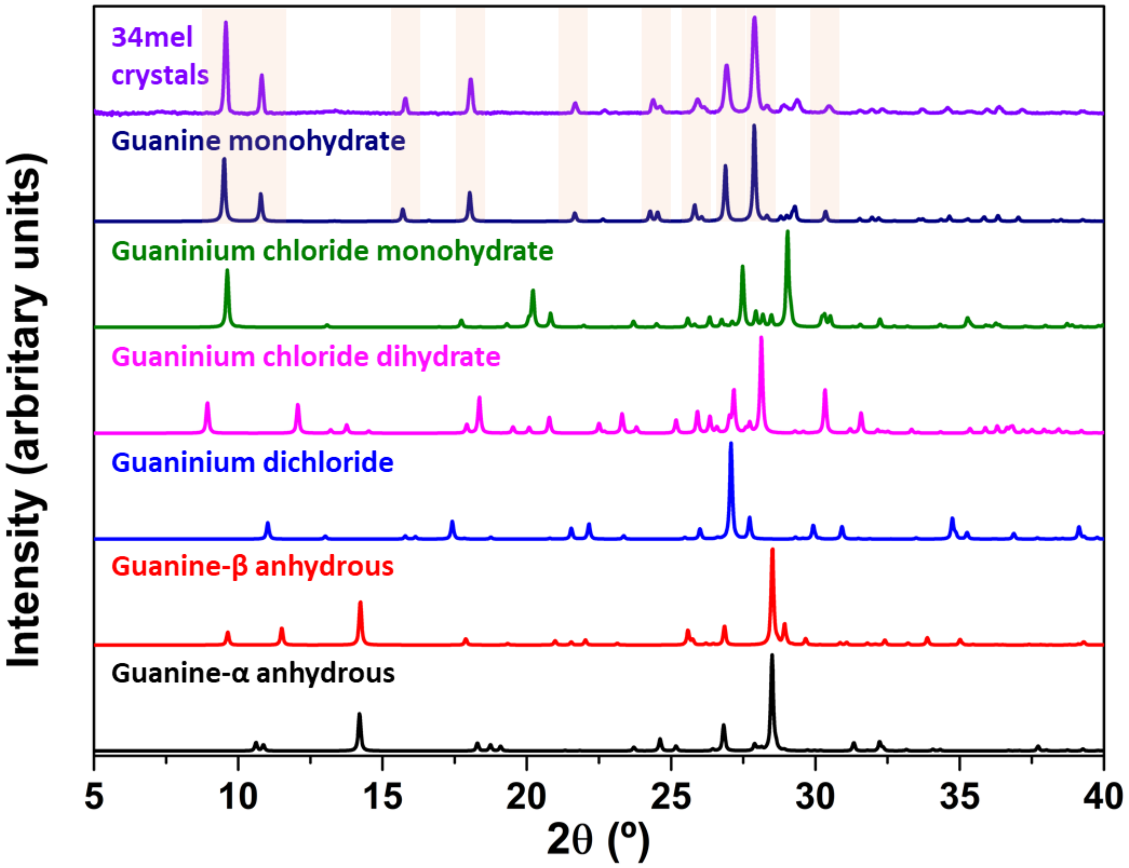

Fig. S7. X-ray diffraction studies of different crystal forms of guanine. Powder X-ray diffraction pattern of the guanine crystals produced by 34mel (violet) and the simulated powder X-ray diffraction pattern obtained from guanine monohydrate (dark blue), guaninium chloride monohydrate (green), guaninium chloride dihydrate (pink), guaninium dichloride (blue), *β* anhydrous guanine (red) and *α* anhydrous guanine (black) single-crystal X-ray data [1-3].

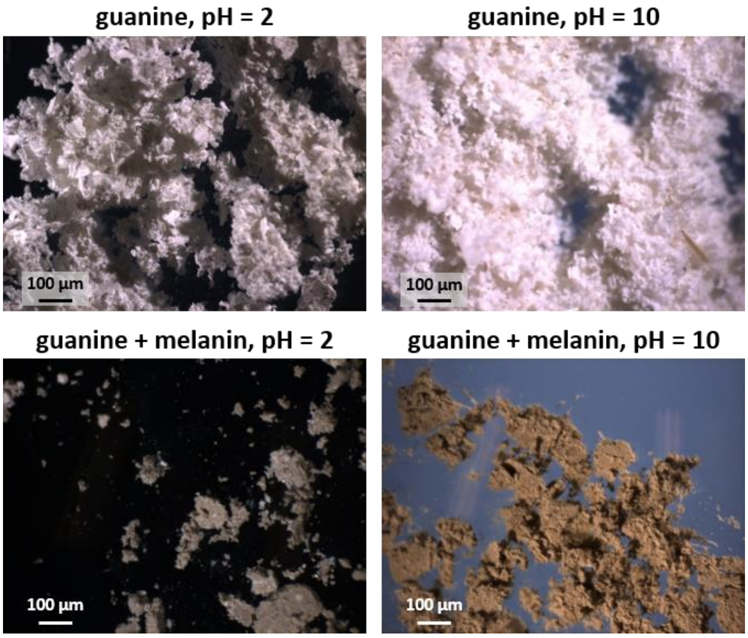

Fig. S8. Crystalline material obtained as result of crystallization experiments of commercial guanine with or without the addition of homogentisate melanin synthesized by 34mel to the solution at different pH conditions. X-ray diffraction patterns of these samples are shown in Additional file 3: Fig. S9.

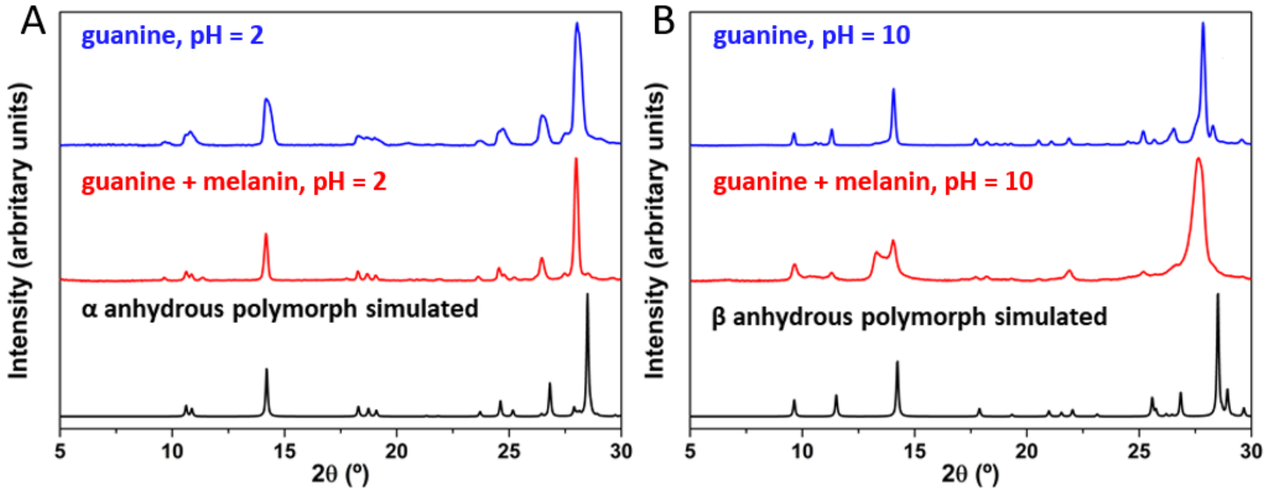

Fig. S9. Powder X-ray diffraction experiments of the crystalline material obtained under different crystallization conditions. Diffraction patterns at pH 2 (**A**) and pH 10 (**B**) for solutions containing commercial guanine with or without melanin produced by 34mel.

Additional methods 2

Single Crystal X-ray diffraction (XRD)

Crystals of guaninium chloride dihydrate crystallized from a solution containing biogenic melanin were studied by single crystal XRD using an Oxford Diffraction Gemini E lab diffractometer with Mo Kα (λ = 0.71 Å) radiation (Additional file 3: Fig. S10). The full data collection was planned using the CrysAlis Pro strategy tool and data was reduced using CrysAlis Pro software (Agilent Technologies, Oxfordshire, UK). A Gaussian method implemented in WinGX [4], or a numerical model [5] was used for the absorption correction. Using Olex2 [6] the structures were solved by Intrinsic phasing employing ShelXL [7] and refined with the ShelXL [8] package using Least Squares minimization. Non-hydrogen atoms were anisotropically refined. Hydrogen atoms were mostly included at geometrically calculated positions with thermal parameters derived from the parent atoms. Hydrogen atoms attached to the water molecules were located on Fourier maps, fixed, and given isotropic displacement parameters depending on the parent atoms. Chloride anions disorder was modeled over two sets of sites with occupancies of 0.26 and 0.74 (Additional file 3: Table S1). Disorder was not modeled for solvent molecules and is represented through large ellipsoids. Additional Crystallographic information is shown in table S1. Crystallographic data for guaninium chloride dihydrate - melanin have been deposited into the Cambridge Structural Database under the deposition number CCDC 2156488.These data can be obtained free of charge from The Cambridge Crystallographic Data Centre via www.ccdc.cam.ac.uk/structures

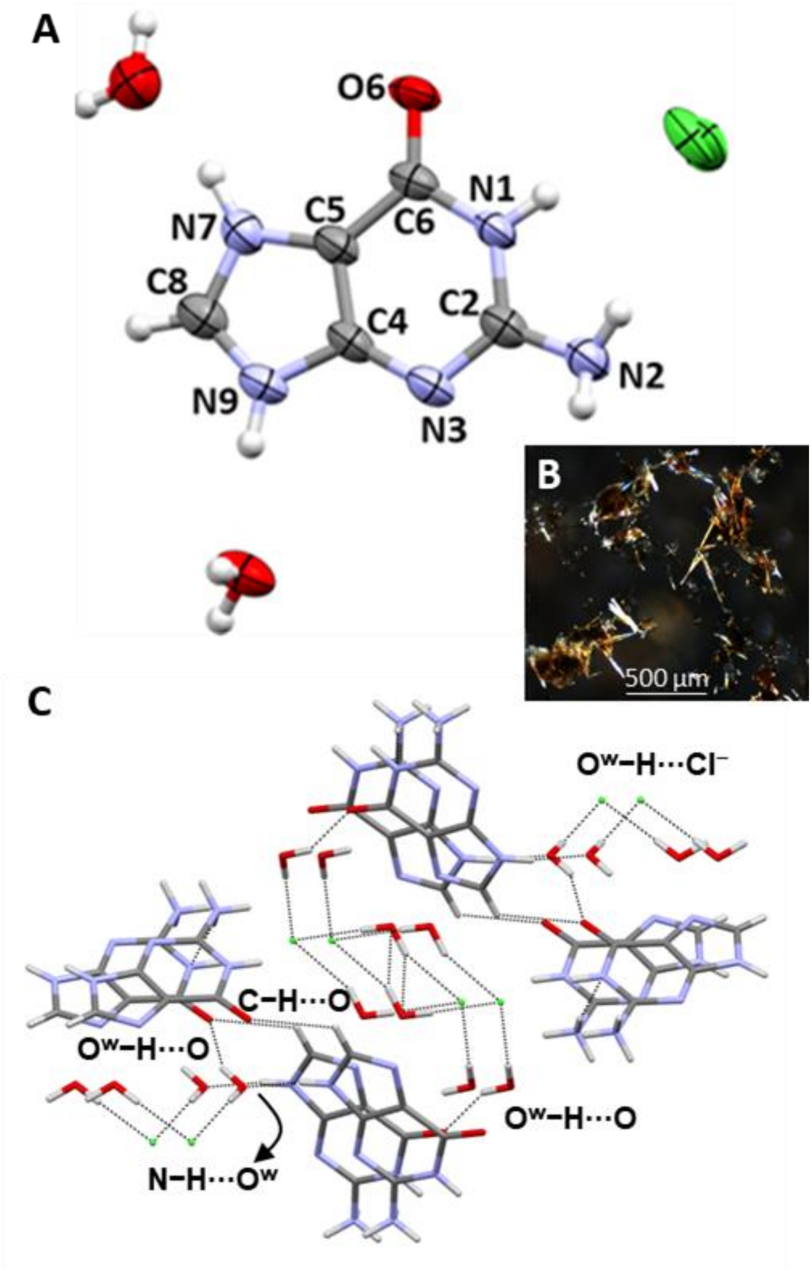

Fig. S10. Structure of guaninium chloride dihydrate crystallized in presence of melanin produced by 34mel determined by single crystal X-ray diffraction. **A** ORTEP plot of the asymmetric unit with displacement ellipsoids drawn at 50% probability level, with numbering. **B** Image of the crystals seen through a polarized light microscope. **C** H-bonded supramolecular network.

Table S1. Crystal data and structure refinement for guaninium chloride dihydrate crystallized in presence of melanin produced by 34mel.

**Crystal data and structure refinement for Guaninium chloride dihydrate - melanin**		**Bond lengths for Guaninium chloride dihydrate - melanin**			**Atomic occupancy for Guaninium chloride dihydrate - melanin**	
		**Atom**	**Atom**	**Length(Å)**	**Atom**	***Occupancy***
Empirical formula	$C_5H_{10}ClN_5O_3$	O1	C2	1.231(5)	Cl1	0.78(5)
Formula weight	223.63	N1	C1	1.370(5)	Cl2	0.22(5)
Temperature/K	298	N1	C2	1.389(6)		
Crystal system	monoclinic	N2	C1	1.336(5)		
Space group	$P2_1/c$	N2	C3	1.334(6)		
a/Å	4.8276(10)	N3	C4	1.373(6)		
b/Å	13.417(2)	N3	C5	1.309(5)		
c/Å	14.6730(14)	N4	C3	1.382(5)		
α/°	90	N4	C5	1.328(6)		
β/°	94.127(14)	N5	C1	1.321(6)		
γ/°	90	C2	C4	1.420(5)		
Volume/$Å^3$	947.9(3)	C3	C4	1.381(6)		
Z	4					
ρ_{calc} g/cm^3	1.567	**Hydrogen bonds for Guaninium chloride dihydrate-melanin**				
μ/mm^{-1}	0.395					
F(000)	464	**D**	**H**	**A**	**d(D-A) (Å)**	**D-H-A (°)**
Crystal size/mm^3	0.1 × 0.05 × 0.05	O2	H2A	O11	2.786(5)	151.9
Radiation	MoKα (λ = 0.71073)	O3	H3B	Cl22	2.95(4)	153.3
2Θ range for data collection/°	8.242 to 57.544	N4	H4	O2	2.687(5)	170.8
Index ranges	-5 ≤ h ≤ 6, -14 ≤ k ≤ 18, -19 ≤ l ≤ 18	N3	H3	O3	2.645(6)	169(4)
Reflections collected	6247	1 + x, 3/2 - y,-½ + z; -1+x, 3/2 - y ,-½ + z				
Independent reflections	2201 [R_{int} = 0.1620, R_{sigma} = 0.1534]					
Data/restraints/parameters	2201/0/148					
Goodness-of-fit on F^2	0.992					
Final R indexes [I ≧ 2σ (I)]	R_1 = 0.0907, wR_2 = 0.2024					
Final R indexes [all data]	R_1 = 0.1606, wR_2 = 0.2638					
Largest diff. peak/hole / e $Å^{-3}$	0.43/-0.49					

Table S2. Comparative analysis of different guaninium chloride dihydrate single crystal X-ray diffraction data

	a	b	c	α	β	γ	Space Group	Ref.
**Guaninium chloride dihydrate** (GUANCD01)	4.8708(7)	13.237(3)	14.638(2)	90	93.906	90	$P2_1/c$	[9]
**Guaninium chloride dihydrate** (GUANCD02)	4.8587(11)	13.228(3)	14.612(3)	90	93.862(4)	90	$P2_1/c$	[10]
**Guaninium chloride dihydrate-melanin**	4.8276(10)	13.417(2)	14.6730(14)	90	94.127(14)	90	$P2_1/c$	this work

Supplementary References

1. Guille K, Clegg W. Anhydrous guanine: a synchrotron study. Acta Cryst. 2006;C62:o515–7.
2. Hirsch A, Gur D, Polishchuk I, Levy D, Pokroy B, Cruz-Cabeza AJ, et al. "Guanigma": The revised structure of biogenic anhydrous guanine. Chem Mater. 2015;27(24):8289–97.
3. Thewalt U, Bugg CE, Marsh RE. The crystal structure of guanine monohydrate. Acta Cryst. 1971;B27:2358–63.
4. Blessing RH. An empirical correction for absorption anisotropy. Acta Cryst. 1995;A51:33–8.
5. Coppens P, Leiserowitz L, Rabinovich D. Calculation of absorption corrections for camera and diffractometer data. Acta Cryst. 1965;18:1035–8.
6. Dolomanov OV, Bourhis LJ, Gildea RJ, Howard JAK, Puschmann H. *OLEX2*: a complete structure solution, refinement and analysis program. J Appl Crystallogr. 2009;42:339–41.
7. Sheldrick GM. Crystal structure refinement with SHELXL. Acta Cryst. 2015;C71:3–8.
8. Sheldrick GM. SHELXT–Integrated space-group and crystal-structure determination. Acta Cryst. 2015;A71:3–8.
9. Bats JW, Grundl MA. CCDC 250428: Experimental crystal structure determination. 2005.
10. Lewis TC, Tocher DA. Redetermination of guaninium chloride dihydrate. Acta Cryst. 2005;E61:o1023–5.
