# Supplementary material for: Guanine crystal formation by bacteria
Source: BMC Biol. 2023 Apr 3;21:66. doi: 10.1186/s12915-023-01572-8 (PMC10071637; doi:10.1186/s12915-023-01572-8)
Supplement: Supplementary file 4 — Additional file 4: Table S3. Bacteria used for genome analysis. [file 12915_2023_1572_MOESM4_ESM.pdf]

Supplementary Information for

Guanine crystal formation by bacteria

María Elisa Pavan, Federico Movilla, Esteban E. Pavan, Florencia Di Salvo, Nancy I. López, M. Julia Pettinari

Additional file 4

Table S3. Bacteria used for genome analysis

Species	GenBank Accession
Aeromonas allosaccharophila CECT 4199^T	NZ_CDBR00000000
Aeromonas aquatica AE235^T	NZ_JRGL01000000
Aeromonas australiensis CECT 8023^T	NZ_CDDH00000000
Aeromonas bestiarum CECT 4227^T	NZ_CDDA00000000
Aeromonas bivalvium CECT 7113^T	NZ_CDBT00000000
Aeromonas caviae CECT 838^T	NZ_JAGDEN000000000.1
Aeromonas dhakensis AAK1	NZ_BAFL00000000
Aeromonas diversa CECT 4254^T	NZ_CDCE00000000.1
Aeromonas encheleia CECT 4342^T	NZ_CDDI00000000
Aeromonas enteropelogenes CECT 4255^T	NZ_CDDE00000000
Aeromonas eucrenophila CECT 4224^T	NZ_CDDF00000000
Aeromonas finlandiensis 4287D^T	NZ_JRGK00000000
Aeromonas fluvialis LMG 24681^T	NZ_CDBO00000000
Aeromonas hydrophila ATCC 7966^T	NC_008570
Aeromonas jandaei CECT 4228^T	NZ_CDBV00000000
Aeromonas lacus AE122^T	NZ_JRGM00000000
Aeromonas media CECT 4232^T	NZ_CDBZ00000000.1
Aeromonas molluscorum 848^T	NZ_AQGQ00000000
Aeromonas piscicola LMG 24783^T	NZ_CDBL00000000
Aeromonas popoffii CIP 105493^T	NZ_CDBI00000000
Aeromonas rivipollensis KN-Mc-11N1	NZ_CP027856.1
Aeromonas rivuli DSM 22539^T	NZ_CDBJ00000000
Aeromonas salmonicida subsp. *achromogenes* AS03	NZ_AMQG00000000.2
Aeromonas salmonicida subsp. *masoucida* NBRC 13784^T	NZ_BAWQ00000000.1
Aeromonas salmonicida subsp. *pectinolytica* 34mel^T	ARYZ00000000.2
Aeromonas salmonicida subsp. *salmonicida* ATCC 33658^T	NZ_CDDW00000000.1
Aeromonas salmonicida subsp. *smithia* JF4097	NZ_JZTI00000000.1
Aeromonas sanarellii LMG 24682^T	NZ_CDBN00000000
Aeromonas schubertii WL1483	NZ_CP013067
Aeromonas simiae CIP 107798^T	NZ_CDBY00000000
Aeromonas sobria CECT 4245^T	CDBW00000000
Aeromonas taiwanensis LMG 24683^T	NZ_BAWK00000000
Aeromonas tecta CECT 7082^T	NZ_CDCA00000000
Aeromonas veronii B565	NC_015424

Escherichia coli K-12 substr. BW25113	NZ_CP009273.1
Pseudomonas aeruginosa PAO1	NC_002516.2
Pseudomonas extremaustralis DSM 25547	AHIP00000000.1
Pseudomonas protegens Pf-5	CP000076.1
Pseudomonas putida KT2440	NC_002947.4
Pseudomonas syringae B728a	NC_007005.1
Shewanella oneidensis MR-1^{T}	NC_004347.2
